# Iota-Carrageenan from Marine Alga *Solieria filiformis* Prevents Naproxen-Induced Gastrointestinal Injury via Its Antioxidant and Anti-Inflammatory Activities

**DOI:** 10.3390/biomedicines12112574

**Published:** 2024-11-10

**Authors:** João L. S. Pinheiro, Willer M. Sousa, Lucas H. M. Rodrigues, Francisco F. Bezerra, Cecília L. O. A. Cunha, Victória M. R. Santos, Samara R. B. D. Oliveira, Rudy D. Bingana, André Luiz. R. Barbosa, Marcellus H. L. P. Souza, Ana Lúcia P. Freitas, Renan O. S. Damasceno

**Affiliations:** 1 Department of Physiology and Pharmacology, Federal University of Pernambuco, Recife 50670-420, PE, Brazil; joao.pinheiro@ufpe.br (J.L.S.P.); lucas.henriquemarques@ufpe.br (L.H.M.R.); cecilialourena08@gmail.com (C.L.O.A.C.); victoria.martins@ufpe.br (V.M.R.S.); 2 Department of Biochemistry and Molecular Biology, Federal University of Ceará, Fortaleza 60020-181, CE, Brazil; willer_biomed@hotmail.com (W.M.S.); felipebezerra_ipu@hotmail.com (F.F.B.); pfreitas@bioquimica.ufc.br (A.L.P.F.); 3 Department of Physiology and Pharmacology, Federal University of Ceará, Fortaleza 60430-275, CE, Brazil; samararodriguesbd@gmail.com (S.R.B.D.O.); binganarudy@hotmail.com (R.D.B.); souzamar.ufc@gmail.com (M.H.L.P.S.); 4 Department of Physiotherapy, Parnaíba Delta Federal University, Parnaíba 64202-020, PI, Brazil; andreluiz@ufpi.edu.br

**Keywords:** iota-carrageenan, *Solieria filiformis*, naproxen, gastrointestinal injury, oxidative stress

## Abstract

**Background:** Non-steroidal anti-inflammatory drugs (NSAIDs) are widely used in therapy due to their anti-inflammatory and analgesic properties. However, their clinical use is often associated with gastrointestinal complications. Thus, this study aimed to investigate the protective effect of a sulfated iota-carrageenan isolated from the marine alga *Solieria filiformis* (IC-Sf) against naproxen-induced gastrointestinal injury. **Methods:** Parameters of gastrointestinal injury, secretory and motor functions, and toxicity were evaluated. **Results:** The results demonstrated that IC-Sf significantly reduced naproxen-induced gastrointestinal macroscopic injury, with a maximum effect observed at 30 mg/kg. IC-Sf also preserved gastrointestinal antioxidant defense and prevented lipid peroxidation, with a reduction in the non-protein sulfhydryl group (NP-SH) and malondialdehyde (MDA) concentrations induced by naproxen. Additionally, IC-Sf mitigated naproxen-induced gastrointestinal inflammation, as evidenced by reduced myeloperoxidase (MPO) activity, tumor necrosis factor-alpha (TNF-α), and interleukin-1 beta (IL-1β). IC-Sf did not alter gastric secretion or gastrointestinal motility. In addition, the animals treated with IC-Sf did not present toxic effects. **Conclusions:** In conclusion, IC-Sf protected the gastrointestinal tract against the harmful effects of naproxen by inhibiting the inflammatory response and lipid peroxidation, suggesting its potential as a new therapeutic agent or food additive.

## 1. Introduction

Marine algae are recognized as promising sources of phenolic compounds, lectins, polysaccharides, and various other bioactive molecules [1,2,3]. The main polysaccharides synthesized by marine algae include ulvans from green algae, fucans from brown algae, and galactans (e.g., agarans and carrageenans) from red algae [4]. These components are predominantly applied in the nutraceutical, cosmeceutical, and pharmacological fields [5,6]. Consequently, due to their potential benefits to human health, both for the prevention and treatment of disease, numerous algae species have attracted the attention of researchers [7,8].

**Figure 1:**
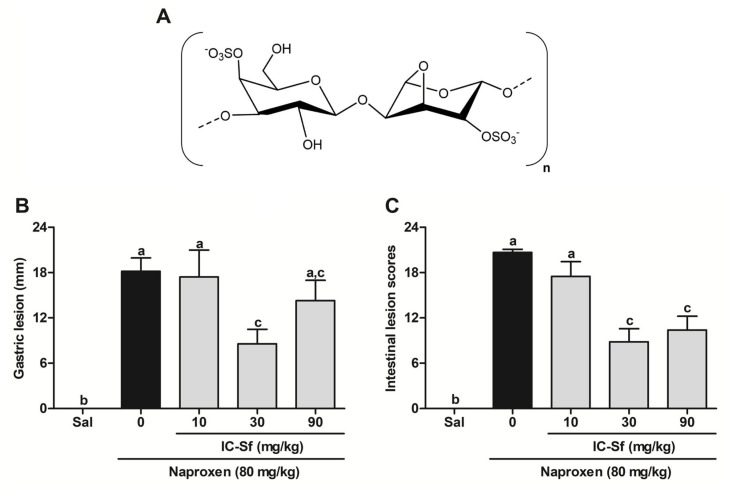
Biochemical structure of iota-carrageenan from *S. filiformis* and its effect against naproxen-induced gastrointestinal macroscopic injury. (**A**) Structure of IC-Sf; (**B**) Gastric and (**C**) Intestinal injury. Different letters (a–c) on the bar represent significant differences between groups (*p* < 0.05).

In this perspective, the red marine alga *Solieria filiformis*, found along the northeast coast of Brazil, mainly in Ceará [9], synthesizes an iota-carrageenan-type polysaccharide [10], which is widely used as a gelling agent [11]. Its chemical structure (Figure 1A) comprises a linear main chain composed of β-D-galactopyranose-4-sulfate and 3,6-anhydro-α-D-galactopyranose-2-sulfate, with a degree of substitution for sulfate groups of 1.08, resulting in a molecular weight of 210.9 kDa [10].

Studies have demonstrated the biological activities of compounds derived from *S. filiformis*, including anti-inflammatory [12], gastroprotective [10], wound healing [13], antidepressant [14], antimicrobial [15], antitumor [16], antiviral, and antioxidant properties [17]. However, research evaluating the efficacy of *S. filiformis* in preventing gastrointestinal lesions through its antioxidant and anti-inflammatory activities as well as its therapeutic safety is scarce in the literature.

NSAIDs, including naproxen, are widely used clinically for their anti-inflammatory and analgesic properties [18]. However, their prolonged use can lead to severe gastrointestinal injuries, characterized by ulcers, hemorrhages, and compromised mucosal integrity [19]. This is attributed to the inhibition of cyclooxygenase 1 (COX-1) and reduced prostaglandin biosynthesis, which protect the gastrointestinal mucosa [20]. Consequently, inflammatory cells are recruited to the injury site, and oxidative stress increases through reactive oxygen species (ROS) and lipid peroxidation [21]. Thus, the identification of safe molecules that enhance gastrointestinal defense mechanisms may attenuate the adverse effects induced by NSAIDs.

The current study aimed to evaluate the protective effect of an iota-carrageenan from the marine alga *S. filiformis* against naproxen-induced gastrointestinal injury as well as its safety profile to fill a gap in the literature. This study’s hypothesis was that the antioxidant and anti-inflammatory effects of this marine alga could play a crucial role in reducing lipid peroxidation, strengthening antioxidant defenses, and preventing inflammation.

## 2. Materials and Methods

### 2.1. Drugs and Reagents

The 5,5′-Dithiobis (2-nitrobenzoic acid) (DTNB), used to determine levels of NP-SH, hexadecyltrimethylammonium bromide (HTAB), used for MPO activity assay, and naproxen, used to induce gastrointestinal injury, were purchased from Sigma-Aldrich (St. Louis, MO, USA). Atropine (Isofarma, Eusébio, CE, Brazil) and Omeprazole (Blau Farmacêutica S.A., Cotia, SP, Brazil) were used as control drugs in the gastrointestinal function evaluation. Ketamine (Sespo Ind. e Com., Paulínia, SP, Brazil) and Xylazine (Syntec, Tamboré, SP, Brazil) were used as anesthetic drugs. Other chemicals and reagents used, such as ethanol (99.5%), KCl, and EDTA, were of analytical grade and were obtained from standard commercial suppliers.

### 2.2. Marine Alga and Iota-Carrageenan

*S. filiformis* was collected at Flexeiras beach (3°13′11.0″ S 39°16′21.5″ W) on the Atlantic coast of Northeast Brazil, Trairí, Ceará. A voucher (No. 56,148) was deposited in the Prisco Bezerra Herbarium of the Federal University of Ceará. For extraction of IC-Sf, 5 g of *S. filiformis* was macerated, suspended in 0.1 M sodium acetate buffer (containing 5 mM EDTA, 5 mM cysteine and papain), incubated for 6 h at 60 °C, filtered, and precipitated with 10% cetylpyridinium chloride. The precipitate was dissolved in NaCl:ethanol (2 M, 100:15, *v*/*v*), precipitated with ethanol and dialyzed extensively using distilled water, and then lyophilized [22]. The biochemical characterization of IC-Sf was performed by microanalysis, Fourier transform infrared (FT-IR) spectroscopy, nuclear magnetic resonance (NMR) spectroscopy, and high-pressure size exclusion chromatography (HPSEC) [10].

### 2.3. Animals

Male Wistar albino rats (150–200 g, *n* = 6) and Balb/c mice (25–30 g, *n* = 6) were housed in a temperature-controlled room (23 ± 2 °C) and maintained under a 12 h light/dark cycle with ad libitum access to water and food. Before the experiments, the animals were fasted for 16–18 h with free access to water. All animal procedures were conducted in accordance with the Guide for Care and Use of Laboratory Animals (National Institutes of Health, Bethesda, MD, USA) and approved by the Ethics Committee on the Use of Animals at the Federal University of Pernambuco (Protocol No. 38/2021).

### 2.4. Experimental Protocols

The rats were divided into five groups: Group I: saline (control), Group II: naproxen, Group III: IC-Sf 10 mg/kg + naproxen, Group IV: IC-Sf 30 mg/kg + naproxen, and Group V: IC-Sf 90 mg/kg + naproxen. IC-Sf was administered orally (0.5 mL/200 g) twice daily for two days. After 1 h, naproxen (80 mg/kg, 0.5 mL/200 g) was administered twice daily, as previously described [23]. The animals were euthanized on the second day, four hours after the final naproxen administration. The stomach and small intestine were excised, opened, washed with saline, and subjected to macroscopic analysis. Tissue samples were frozen at −80 °C for analysis of non-protein sulfhydryl groups (NP-SH), malondialdehyde (MDA), myeloperoxidase (MPO), interleukin-1β (IL-1β), and tumor necrosis factor-α (TNF-α).

### 2.5. Macroscopic Injury

For gastric injury, the stomach was opened along the greater curvature and washed with saline. The area of the injury was measured using a digital caliper (Mitutoyo^®^, Kawasaki, Japan) and expressed in millimeters (mm). The intestinal injury was evaluated in a 5 cm portion of the isolated small intestine, washed with saline, fixed in a plastic block, and analyzed based on established criteria [24].

### 2.6. NP-SH Levels

Tissue samples were homogenized in cold EDTA. The homogenate was mixed with 50% TCA and centrifuged at 3000 rpm for 15 min. Tris buffer (0.4 M, pH 8.9) and DTNB were added to the supernatant and stirred for 3 min, and absorbance was measured at 412 nm. The results are expressed in micrograms of NP-SH per gram of tissue (µg/g of tissue) [25].

### 2.7. MDA Concentration

The samples were homogenized in cold KCl buffer. The homogenate was mixed with 1% H_3_PO_4_ and 0.6% tert-butyl alcohol. This mixture was stirred and heated in a boiling water bath for 45 min. The preparation was cooled in an ice water bath, followed by the addition of n-butanol. This mixture was then stirred, and the butanol was removed by centrifugation at 1200 rpm for 15 min. The absorbance was measured at 520 and 535 nm. The results are expressed in nanomoles of MDA per gram of tissue (nmol/g of tissue) [26].

### 2.8. MPO Activity

The tissue samples were homogenized in 0.5% HTAB. The homogenate was centrifuged at 4500 rpm for 20 min, and MPO activity was evaluated at 450 nm using o-dianisidine and 1% H_2_O_2_. The results are expressed in units of MPO per milligram of tissue (U/mg of tissue) [27].

### 2.9. Cytokine Levels

The rat IL-1β and TNF-α levels were determined using an enzyme-linked immunosorbent assay (ELISA) with commercial kits, according to the manufacturer’s instructions (DuoSet ELISA Development kit R&D Systems, Minneapolis, MN, USA). The results are expressed in picograms of cytokine per gram of tissue (pg/g of tissue) [28].

### 2.10. Gastric Secretion

The mice were divided into three groups: Group I: saline (control), Group II: IC-Sf 30 mg/kg (0.5 mL/25 g), and Group III: omeprazole (a gastric acid secretion inhibitor; 40 mg/kg, i.p.). The animals were anesthetized with ketamine *+* xylazine (80 mg/kg and 8 mg/kg, i.p., respectively), and an incision was performed in the epigastric region to locate the stomach, with the pylorus tied with thread. The abdominal wall was sutured, and four hours post-surgery, the animals were euthanized, and their stomachs were removed for gastric juice collection. Volume, total acidity, pH, and peptic activity were measured [29].

### 2.11. Gastric Emptying and Gastrointestinal Transit

The mice received IC-Sf (30 mg/kg; 0.5 mL/25 g), saline, or atropine (a cholinergic antagonist used to decrease gastrointestinal motility; 3 mg/kg, s.c.). One hour later, phenol red (a non-absorbable marker; 0.75 mg/mL) was administered. After 20 min, the animals were euthanized; a laparotomy was performed; and the gastroesophageal, gastroduodenal, and ileocecal junctions were isolated and removed, dividing them into segments for the stomach and small intestine (I1 to I5). Each segment was homogenized in 0.1 N NaOH and centrifuged 20 min later. The samples were mixed with TCA and centrifuged again. The supernatant was added to 0.5 N NaOH, and absorbance was measured at 560 nm. The results of gastric emptying are expressed in percentage (%) of fractional dye retention [30]. Gastrointestinal transit was estimated according to the geometric center [31], with the sum of the values of each segment (stomach and I1 to I5) indicated by the geometric center of the marker propelled along the gastrointestinal tract, aligning with the center of mass of objects.

### 2.12. Acute Toxicity

The mice treated with IC-Sf (2000 mg/kg, p.o.; 0.5 mL/25 g) or saline were observed for 14 days [32]. Body mass and behavioral changes (Hippocratic screening) were evaluated [33]. Blood samples were collected for biochemical analysis, and key organs (kidneys, spleen, liver, heart, and lungs) were collected for macroscopic and histopathological analyses.

#### 2.12.1. Macroscopic and Histological Analyses

The organs were removed for macroscopic evaluation of color, shape, and size. Then, the organs were photographed, weighed, and collected for histological analysis. For this, the samples were fixed in 10% formaldehyde, processed, and embedded in paraffin. Sections with a thickness of 4 μm were placed on slides, stained with hematoxylin and eosin (HE), and evaluated [34].

#### 2.12.2. Biochemical Analysis

Blood samples were collected by puncture in the retro-orbital plexus, placed in tubes containing anticoagulant, and centrifuged at 3500 rpm for 10 min. Alkaline phosphatase, aspartate aminotransferase (AST), alanine aminotransferase (ALT), albumin, glucose, total cholesterol, urea, and creatinine were analyzed using biochemical kits, according to the manufacturer’s instructions (Labtest^®^, Lagoa Santa, MG, Brazil) [34].

### 2.13. Statistical Analysis

The results are expressed as the mean ± standard error of the mean (SEM). Statistical differences between means of different groups were analyzed using one-way analysis of variance (ANOVA), followed by the Duncan multiple range test. Acute toxicity data were compared using the *t*-test. Differences were considered statistically significant when *p* < 0.05 [35].

## 3. Results

### 3.1. IC-Sf Prevents Naproxen-Induced Gastrointestinal Injury

The protective effect of IC-Sf against naproxen-induced gastrointestinal injury in rats was evaluated through macroscopic assessment. Naproxen administration (18.2 ± 1.8 mm; Figure 1B) resulted in intense gastric lesions. However, IC-Sf administration at doses of 10, 30, or 90 mg/kg mitigated these effects (lesion sizes of 17.4 ± 3.6 mm, 8.6 ± 1.9 mm, and 14.3 ± 2.7 mm, respectively), with maximal and significant effects (*p* < 0.05) observed at 30 mg/kg. Naproxen also induced macroscopic injury in the small intestine (20.7 ± 0.4 lesion score; Figure 1C), which was significantly (*p* < 0.05) attenuated by IC-Sf treatment (lesion scores of 17.5 ± 2.0, 8.8 ± 1.7, and 10.4 ± 1.8 for 10, 30, and 90 mg/kg, respectively). Because of its efficacy, the 30 mg/kg dose of IC-Sf was selected for subsequent experiments.

### 3.2. IC-Sf Prevents Naproxen-Induced Gastrointestinal Oxidative Imbalance

**Figure 2:**
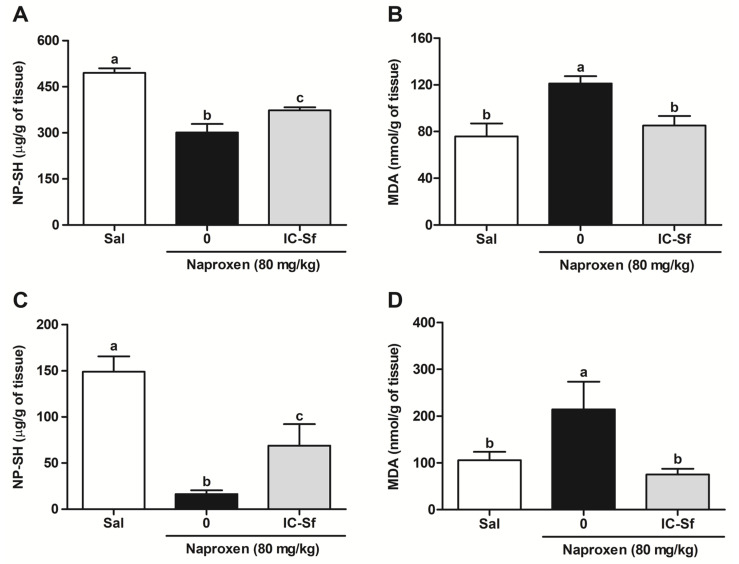
Effect of IC-Sf on naproxen-induced gastrointestinal oxidative stress. (**A**) NP-SH levels and (**B**) MDA concentration in the gastric mucosa; and (**C**) NP-SH levels and (**D**) MDA concentration in small intestine mucosa. Different letters (a–c) on the bar represent significant differences between groups (*p* < 0.05).

To assess whether the protective effect of IC-Sf against naproxen-induced gastrointestinal injury in rats involved control of oxidative stress, tissue NP-SH levels and MDA concentration were analyzed. Naproxen significantly (*p* < 0.05) increased NP-SH consumption in gastric (301.1 ± 27.5 µg/g of tissue; Figure 2A) and small intestine mucosa (16.4 ± 4.2 µg/g of tissue; Figure 2C) compared to the control (495.4 ± 15.1 µg/g of tissue and 149.0 ± 16.7 µg/g of tissue, respectively). IC-Sf administration restored NP-SH levels (373.3 ± 10.0 µg/g of tissue and 69.0 ± 23.3 µg/g of tissue, respectively) in both tissues. Additionally, MDA concentration in the gastric and small intestine mucosa significantly (*p* < 0.05) increased after naproxen administration (121.1 ± 6.4 µg/g of tissue and 214.5 ± 58.8 µg/g of tissue, respectively; Figure 2B,D) compared to the control (75.8 ± 11.2 µg/g of tissue and 105.8 ± 17.9 µg/g of tissue, respectively), whereas IC-Sf treatment significantly (*p* < 0.05) reduced MDA concentration (85.0 ± 8.3 µg/g of tissue and 75.3 ± 12.2 µg/g of tissue, respectively) altered by naproxen.

### 3.3. IC-Sf Reduces Naproxen-Induced Gastrointestinal Inflammatory Response

**Figure 3:**
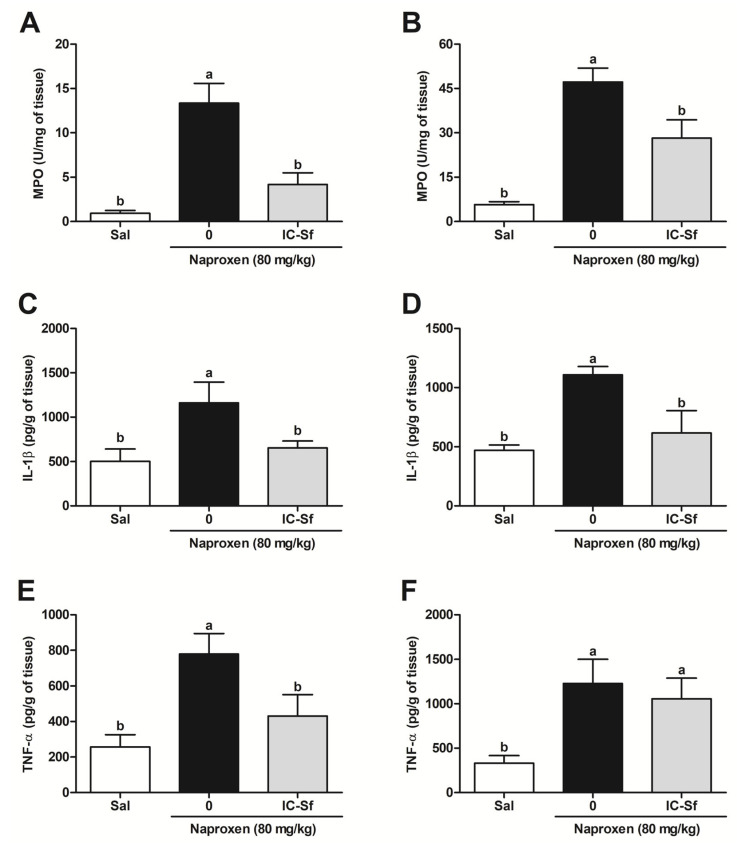
Effect of IC-Sf on naproxen-induced gastrointestinal inflammation. (**A**,**B**) MPO activity; (**C**,**D**) IL-1β levels; (**E**,**F**) TNF-α levels. Different letters (a,b) on the bar represent significant differences between groups (*p* < 0.05).

MPO activity, a marker of neutrophilic infiltration, was assessed in rat tissue to determine if IC-Sf mitigates the influx of naproxen-induced inflammatory cells. Compared to the control group, naproxen significantly (*p* < 0.05) increased MPO activity in both gastric (13.3 ± 2.2 U/mg of tissue versus 0.9 ± 0.3 U/mg of tissue; Figure 3A) and small intestine mucosa (47.2 ± 4.7 U/mg of tissue versus 5.7 ± 1.0 U/mg of tissue; Figure 3B). IC-Sf treatment significantly (*p* < 0.05) inhibited this increase in both tissues (4.2 ± 1.3 U/mg of tissue and 28.2 ± 6.1 U/mg of tissue, respectively).

Next, the role of IC-Sf on cytokine levels in the gastrointestinal tissue was investigated. Compared to the control group, naproxen significantly (*p* < 0.05) increased IL-1β levels in the gastric (1163.0 ± 233.1 pg/mL versus 502.6 ± 140.0 pg/mL; Figure 3C) and small intestine mucosa (1108.0 ± 69.6 pg/mL versus 469.3 ± 46.4 pg/mL; Figure 3D). However, IC-Sf reduced IL-1β levels in both tissues (652.8 ± 79.3 pg/mL and 616.8 ± 188.8 pg/mL, respectively). Naproxen significantly (*p* < 0.05) elevated TNF-α levels in the gastric (779.9 ± 115.9 pg/mL; Figure 3E) and small intestine mucosa (1228.0 ± 273.6 pg/mL; Figure 3F) compared to the control group (257.5 ± 68.1 pg/mL and 329.3 ± 87.2 pg/mL, respectively). However, IC-Sf administration significantly (*p* < 0.05) suppressed this increase in the gastric mucosa (430.4 ± 121.2 pg/mL) but not in the small intestine mucosa (1055.01 ± 231.9 pg/mL).

### 3.4. IC-Sf Does Not Alter Gastric Secretion, Gastric Emptying, or Gastrointestinal Transit

To assess the impact of IC-Sf on physiological functions, gastrointestinal secretion and motility were evaluated in the mice. Compared to the control group, IC-Sf did not significantly alter gastric parameters, such as fluid volume (128.0 ± 25.5 µL versus 109.8 ± 20.3 µL), total acidity (0.037 ± 0.002 Eq[H+]/mL/4 h versus 0.034 ± 0.002 Eq[H+]/mL/4 h), pH (1.42 ± 0.02 versus 1.47 ± 0.02), or pepsin activity (622.9 ± 20.4 µM of tyrosine/4 h versus 587.5 ± 19.6 µM of tyrosine/4 h; Table 1). In contrast, omeprazole, a positive control, significantly affected these parameters, observed through reduced volume (52.7 ± 2.3 µL), decreased total acidity (0.021 ± 0.005 Eq[H+]/mL/4 h), increased pH (1.71 ± 0.09), and reduced pepsin activity (531.7 ± 13.9 µM of tyrosine/4 h). Additionally, IC-Sf did not significantly alter gastric emptying (24.0 ± 2.2% versus 25.9 ± 3.0% of retention; Table 1), whereas atropine, a positive control, significantly (*p* < 0.05) increased gastric retention (36.8 ± 2.8%). Compared to the control, IC-Sf did not alter gastrointestinal transit (2.48 ± 0.05 versus 2.55 ± 0.06) but was significantly (*p* < 0.05) reduced by atropine (1.87 ± 0.07).

### 3.5. IC-Sf Does Not Cause Acute Toxic Effects

**Figure 4:**
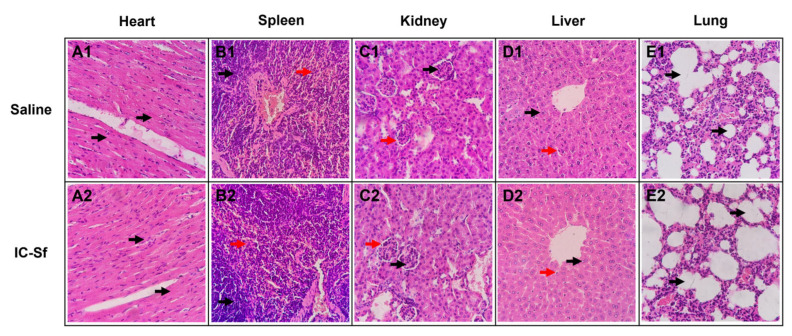
Photomicrograph of organs. Heart (**A**), spleen (**B**), kidney (**C**), liver (**D**), and lung (**E**) of control (**A1**–**E1**) or IC-Sf-treated (**A2**–**E2**) animals stained with hematoxylin and eosin (HE). The analysis revealed no differences between groups. The heart presented normal cardiac muscle fibers (arrows), and it was possible to distinguish the white pulp (black arrows) and the red pulp (red arrows) in the spleen. The renal glomeruli (black arrows) and Bowman’s space (red arrows) presented the expected conformation. In the liver, there were no morphological alterations in hepatocytes (black arrows) or Kupffer cells (red arrows), while well-preserved alveolar structures were observed in the lungs (arrows). Tissue sections were observed under a light microscope at 400×.

Toxicological analyses were performed in mice to evaluate the safety profile of IC-Sf and validate its beneficial effects. The data revealed no significant differences in body mass variation between the animals that received IC-Sf (+10.8 ± 2.4%) and the control (+8.0 ± 1.3%). Compared to the control group, IC-Sf did not significantly alter relative weights of the heart (0.146 ± 0.016 versus 0.141 ± 0.009 g/25 g), spleen (0.110 ± 0.004 versus 0.100 ± 0.004 g/25 g), kidneys (0.387 ± 0.009 versus 0.411 ± 0.018 g/25 g), liver (1.210 ± 0.042 versus 1.290 ± 0.051 g/25 g), or lungs (0.146 ± 0.004 versus 0.154 ± 0.014 g/25 g). Furthermore, the histological analysis of key organs did not reveal any structural abnormalities or cellular lesions in the animals treated with IC-Sf or saline (Figure 4A1–E1,A2–E2).

Compared to the control group, IC-Sf also did not significantly alter serum levels of albumin (2.2 ± 0.1 g/dL versus 2.1 ± 0.2 g/dL), glucose (117.4 ± 8.9 mg/dL versus 111.0 ± 3.2 mg/dL), total cholesterol (120.7 ± 5.7 mg/dL versus 123.5 ± 8.0 mg/dL), ALT (44.1 ± 2.4 U/L versus 45.6 ± 4.0 U/L), AST (86.3 ± 4.4 U/L versus 85.5 ± 3.5 U/L), alkaline phosphatase (59.6 ± 3.6 U/L versus 57.5 ± 5.8 U/L), urea (50.0 ± 3.2 mg/dL versus 53.4 ± 6.2 mg/dL), or creatinine (0.30 ± 0.04 mg/dL versus 0.30 ± 0.03 mg/dL; Table 2).

## 4. Discussion

The current study demonstrated the protective effect of a sulfated iota-carrageenan from the marine alga *S. filiformis* against naproxen-induced gastrointestinal injury in rodents. This macromolecule effectively reduced the gastric and intestinal injury induced by naproxen, preserving redox balance, reducing NP-SH consumption and MDA concentration, and decreasing inflammation, as measured by MPO activity and TNF-α and IL-1β levels. Additionally, this iota-carrageenan does not alter gastrointestinal physiological functions and does not present acute toxicity.

Our research group has previously chemically and structurally characterized this sulfated iota-carrageenan from *S. filiformis* and demonstrated its antioxidant activity and protective effects against alcoholic gastropathy [10]. Other studies have also shown its bioactive properties, including anti-inflammatory and antinociceptive [36,37], and vascular effects [38]. Despite its therapeutic potential, more studies on this iota-carrageenan are necessary, particularly investigations into disorders involving inflammatory and oxidative components as well as their safety profile for future biomedical applications.

Initially, the effect of this macromolecule against naproxen-induced gastrointestinal injury was investigated. NSAIDs cause severe toxicity in the gastrointestinal tract due to their ability to reduce endogenous prostaglandin (PGs) levels, activate neutrophils, and generate reactive oxygen species (ROS) [39]. Therefore, the search for bioactive molecules is essential for the development of new therapeutic strategies for digestive disorders. The results of the current study demonstrated that a sulfated iota-carrageenan from *S. filiformis* reduced naproxen-induced gastric and intestinal injury, demonstrating a maximum effect at 30 mg/kg, thereby highlighting its protective role in the gastrointestinal tract. Similar findings were observed in a previous study carried out by our research group using a sulfated polysaccharide from *Gracilaria birdie*, where the blockade of the redox imbalance induced by naproxen was crucial for its protective effects [40].

Gastrointestinal homeostasis involves a dynamic balance between mucosa protective and aggressive factors, where antioxidant defense plays a crucial role in scavenging free radicals and preventing lipid peroxidation [41]. Free radicals are harmful species implicated in the pathophysiology of various digestive disorders, such as ethanol-induced gastropathy [42], alendronate-induced gastric damage [43], TNBS-induced colitis [44], and chemotherapy-induced intestinal mucositis [45]. Therefore, the evaluation of oxidative stress parameters is essential to elucidate the protective effects of iota-carrageenan against naproxen-induced gastrointestinal injury.

In this context, naproxen-induced oxidative stress was evaluated in the gastrointestinal tract using two markers: NP-SH and MDA. NP-SH is abundant in gastrointestinal tissue, acting as a free radical scavenger [46], while MDA is a product of lipid peroxidation and is widely used as marker of oxidative injury. The findings of the current study indicate that iota-carrageenan from *S. filiformis* preserved NP-SH consumption and reduced MDA concentration in the gastric and small intestine mucosa induced by naproxen, suggesting its key role in regulating oxidative stress for gastrointestinal protection. Similar results were obtained with iota-carrageenan from the red marine alga *Agardhiella ramosissima* in an experimental model of ethanol-induced gastropathy, which demonstrates the antioxidant potential of these molecules [47]. Another possibility is that increased NP-SH levels may be secondary to decreased free radical production. Moreover, in vitro studies have demonstrated the high ability of this iota-carrageenan to chelate and scavenge free radicals [10,17], suggesting antioxidant activity associated with its protective effect on the gastrointestinal tract.

Neutrophil infiltration is also crucial in gastrointestinal complications resulting from NSAIDs such as naproxen, as this process can alter microcirculation and generate toxic free radicals in the mucosa [48]. The inhibition of prostaglandin synthesis and activation of neutrophils by naproxen contribute to the inflammatory response and gastrointestinal lesions [49]. In this sense, MPO, an enzyme present in the azurophilic granules of neutrophils, is released into the extracellular environment after the recruitment of these cells to the inflamed tissue and used as a marker for neutrophilic infiltrate [50]. In turn, sulfated polysaccharides from marine algae are already known for their immunomodulating activity [51], which may be useful for controlling inflammation. In fact, an agaran from *G. birdiae* has been shown to reduce leukocyte recruitment to the inflammation site, contributing to gastrointestinal protection against the harmful effects of naproxen [40]. The findings of the current study showed that iota-carrageenan from *S. filiformis* reduced MPO activity in the gastric and small intestine mucosa in response to the harmful effects of naproxen on the gastrointestinal tract, indicating an inhibitory effect on inflammatory cell migration.

The recruitment of inflammatory cells to the gastrointestinal mucosa is mediated by pro-inflammatory cytokines, such as TNF-α and IL-1β, which play a critical role in inflammation progression [52]. These cytokines act as chemotactic agents, mediating neutrophil recruitment and activation and, thus, contributing to tissue injury through the production of pro-inflammatory mediators and free radicals [53]. A marked reduction in the levels of pro-inflammatory cytokines has already been demonstrated using fucoidan from the brown marine alga *Kjellmaniella crassifolia* in a model of aspirin-induced gastric injury, which supports the important anti-inflammatory activity of these polysaccharides [54]. In this line, the iota-carrageenan from *S. filiformis* reduced TNF-α and IL-1β levels in the gastric and small intestinal mucosa, indicating its essential role in controlling tissue inflammation. These results corroborate with the previously observed reduction in MPO activity and demonstrate the importance of this iota-carrageenan as a protective agent of the gastrointestinal tract.

Additionally, it was investigated whether iota-carrageenan from *S. filiformis* regulates gastric acid secretion as part of its gastrointestinal protective effects. Hydrochloric acid secreted by gastric parietal cells creates an acidic environment (pH < 2) that kills food-derived bacteria and facilitates digestion and the absorption of nutrients [55]. However, excessive acid secretion can result in a breach of the epithelial barrier and gastrointestinal injury. Thus, molecules that reduce acid secretion can attenuate the damage to the gastrointestinal mucosa induced by aggressive agents [56]. Proton pump inhibitors (PPI), including omeprazole, reduce gastric acid secretion by inhibiting H⁺/K⁺ ATPase of gastric parietal cells [57] but lack other protective effects on the gastrointestinal mucosa. The data indicate that iota-carrageenan from *S. filiformis* does not affect gastric secretory parameters, such as fluid volume, total acidity, pH, or pepsin activity, suggesting that its gastrointestinal protection is independent of a gastric antisecretory effect. On the other hand, this macromolecule is recognized for its antioxidant and anti-inflammatory properties, which corroborates the better therapeutic effects in the gastrointestinal tract.

Additionally, gastric emptying and gastrointestinal transit were evaluated to investigate whether iota-carrageenan alters motility. Gastrointestinal motor function is essential to promote the movement of luminal contents and facilitate digestion and absorption as well as the removal of undigested residues [58]. The results of the current study demonstrated that iota-carrageenan from *S. filiformis* does not affect gastric emptying or gastrointestinal transit, indicating no impact on motility.

Next, it was evaluated whether iota-carrageenan from *S. filiformis* has acute toxic effects. Polysaccharides from marine algae exhibit diverse biological activities that make them promising for therapeutic use. Thus, preclinical toxicity studies play a fundamental role in determining the safety profile of new bioactive compounds and represent a crucial point for application in biomedical sciences [59]. The results did not reveal lethality or changes in behavioral parameters in the animals that received iota-carrageenan from *S. filiformis*. These data were associated with the maintenance of body mass, suggesting no interference with macronutrient metabolism.

Changes in organ systems are reliable signs of tissue injury caused by toxic substances [60]. In the current study, macroscopic and histopathological evaluations of the heart, spleen, kidneys, liver, and lungs of the animals treated with iota-carrageenan were performed. No alterations were observed in the location, shape, size, color, or texture of the organs. Furthermore, relative weight analysis indicated that iota-carrageenan did not negatively affect the organs, implying the absence of toxic effects. These results were corroborated by the histopathological analysis, which did not reveal tissue lesions or alterations in structural organization following iota-carrageenan administration. These evaluations are crucial for understanding potential tissue changes in toxicity studies [34,61]. Thus, the data suggest that iota-carrageenan from *S. filiformis* does not induce acute toxic effects on the major organs of animals.

Acute toxic effects can lead to changes in serum levels of albumin, glucose, or total cholesterol, indicating serious adverse conditions, including liver dysfunction, malnutrition, systemic inflammation, or alterations in carbohydrate and lipid metabolism [62]. Additionally, markers such as ALT, AST, and alkaline phosphatase are crucial for assessing hepatotoxicity, with elevations often exceeding three times the baseline level [63]. Similarly, elevated levels of urea and creatinine in blood may indicate potential toxic effects on kidney cells, associated with increased protein catabolism or, more critically, reduced glomerular filtration rate and renal clearance [64]. However, this study’s results demonstrated that serum biomarker values were unchanged in the animals that received iota-carrageenan from *S. filiformis*, supporting its lack of acute toxicity.

## 5. Conclusions

Sulfated iota-carrageenan from the marine alga *S. filiformis* exhibited a protective effect against naproxen-induced gastrointestinal injury by reducing lipid peroxidation, as observed through MDA concentration, preserving antioxidant defenses, as shown by NP-SH levels, and preventing inflammatory cell migration, as evidenced by MPO activity. Furthermore, this macromolecule did not alter gastric secretion or gastrointestinal motility and did not induce systemic toxic effects. The results support the potential use of this iota-carrageenan as a promising tool in the management of digestive disorders. Further studies are necessary to understand the structure–activity relationship and pharmacokinetic parameters of these macromolecules.

## Figures and Tables

**Table 1:** Effect of IC-Sf on gastrointestinal physiological response.

Groups	Gastric Secretion				Motility	
	Volume (µL)	Total Acidity (Eq[H+]/mL/4 h)	pH	Pepsin Activity (µM Tyrosine/4 h)	Gastric Emptying (% Retention)	Gastrointestinal Transit
Saline	109.8 ± 20.3 ^a^	0.034 ± 0.002 ^a^	1.47 ± 0.02 ^b^	587.5 ± 19.6 ^a^	25.9 ± 3.0 ^b^	2.55 ± 0.06 ^a^
IC-Sf	128.0 ± 25.5 ^a^	0.037 ± 0.002 ^a^	1.42 ± 0.02 ^b^	622.9 ± 20.4 ^a^	24.0 ± 2.2 ^b^	2.48 ± 0.05 ^a^
Omeprazole	52.7 ± 2.3 ^b^	0.021 ± 0.005 ^b^	1.71 ± 0.09 ^a^	531.7 ± 13.9 ^b^	0	0
Atropine	0	0	0	0	36.8 ± 2.8 ^a^	1.87 ± 0.07 ^b^

Data are expressed as mean ± SEM. Different letters (a,b) on the bar represent significant differences between groups (*p* < 0.05).

**Table 2:** Biochemical analysis.

Parameter	Saline	IC-Sf
Albumin (g/dL)	2.1 ± 0.2	2.2 ± 0.1
Glucose (mg/dL)	111.0 ± 3.2	117.4 ± 8.9
Total Cholesterol (mg/dL)	123.5 ± 8.0	120.7 ± 5.7
ALT (U/L)	45.6 ± 4.0	44.1 ± 2.4
AST (U/L)	85.5 ± 3.5	86.3 ± 4.4
Alkaline Phosphatase (U/L)	57.5 ± 5.8	59.6 ± 3.6
Urea (mg/dL)	53.4 ± 6.2	50.0 ± 3.2
Creatinine (mg/dL)	0.30 ± 0.03	0.30 ± 0.04

Data are expressed as mean ± SEM (*n* = 3).

## Data Availability

The data presented in this study will be made available on request from the corresponding author.
